# A Delayed Splenic Tragedy Beyond the Scope of a Colonoscope: A Rare Find

**DOI:** 10.7759/cureus.7805

**Published:** 2020-04-23

**Authors:** Munis M Ahmed, Zarak H Khan, Syeda Ramsha Zaidi, Kashif Mukhtar

**Affiliations:** 1 Internal Medicine, St. Mary Mercy Hospital, Livonia, USA; 2 Internal Medicine, King Edward Medical University, Lahore, PAK

**Keywords:** splenic trauma, spleen, rupture, hemorrhage, colonoscopy, complication, blood thinner, apixaban, atrial fibrillation

## Abstract

Colonoscopy is considered a low-risk procedure worldwide. Complications include hemorrhage, bowel perforation, and splenic rupture on rare occasions. The incidence of splenic rupture estimates between 0.00005 and 0.017%. Due to its nonspecific presentation, many cases may be misdiagnosed. We present a 76-year-old female on apixaban for atrial fibrillation who presented to us with sudden-onset, left-sided atraumatic chest pain radiating to the left shoulder, 10/10 in intensity, associated with nausea. The patient underwent an uncomplicated colonoscopy 16 days earlier with the excision of a 1.3 cm polyp. On presentation, her blood pressure was 96/58 mmHg, hemoglobin of 7.2, an international normalized ratio (INR) of 1.6. An abdominal computed tomography scan showed findings suspicious for splenic rupture. In the emergency department, two packed red blood cells (PRBCs) were transfused, and the patient was shifted to the operating room for emergent exploratory laparotomy where a splenectomy was performed for splenic rupture. The patient was discharged six days later without any postoperative complications. We speculate the use of apixaban and our patient's unusual site of pain following splenic rupture to be somehow correlated.

## Introduction

Every procedure is an intervention with its downsides, and as physicians, we should keep this in mind whenever we order one. Nevertheless, modern-day medicine relies on these investigations for absolute patient care. Guidelines come into play as an ever-developing tool where the risks of this procedure outweigh the benefits, focusing on the indications and contraindications. While some of them can be classified as low-risk, others are considered high-risk. Colonoscopy is thought to have a low-risk profile and is commonly performed worldwide with an estimated mortality rate of around 2.9 per 100,000 procedures [1]. Even though colonoscopy is considered safe, complications can still arise. These include hemorrhage and bowel perforation with a reported incidence of 1% and 0.1%, respectively [2]. A colonoscope is a rigid-flexible tube with a camera at the tip for visualization of the gastrointestinal tract. The scope is advanced as per the judgment of the gastroenterologist and based on the need to assess the extent of the disease. Even though it stays inside the lumen of the gut, minor hits to the surrounding viscera are expected. One organ a gastroenterologist should be wary of is the spleen which lies in close proximity to the transverse and the descending colon. Wherry and Zehner reported the first-ever case of splenic injury following colonoscopy in 1974 [3]. Its incidence is estimated to be between 0.00005% and 0.017% [4]. However, due to the nonspecific presentation of splenic injury, many cases may be misdiagnosed and up to 11% of these patients are discharged with different diagnoses [5]. Therefore, the actual incidence of the disease is higher than expected [6-7]. We describe the case of a 76-year-old female with a spleen rupture 16 days after a routine colonoscopy. 

This case was also presented at the American College of Gastroenterology 2019 Annual Scientific Mtg, San Antonio, TX, Oct. 27, 2019: Khan ZH, Korpole PR, Jarodiya V, Singh T, Sultani N: P0154 - Left-sided chest pain following colonoscopy: think outside the colon! (http://www.eventscribe.com/2019/ACG/fsPopup.asp?Mode=presenterInfo&PresenterID=705201).

## Case presentation

A 76-year-old female with a past medical history of atrial fibrillation on apixaban, coronary artery disease, recurrent bladder cancer, and chronic obstructive pulmonary disease presented for sudden onset of chest pain. The pain was 10/10 in intensity and located in the left anterior chest wall region with radiation to the left shoulder. The patient also had associated nausea without vomiting. She denied any history of trauma.

**Figure 1:**
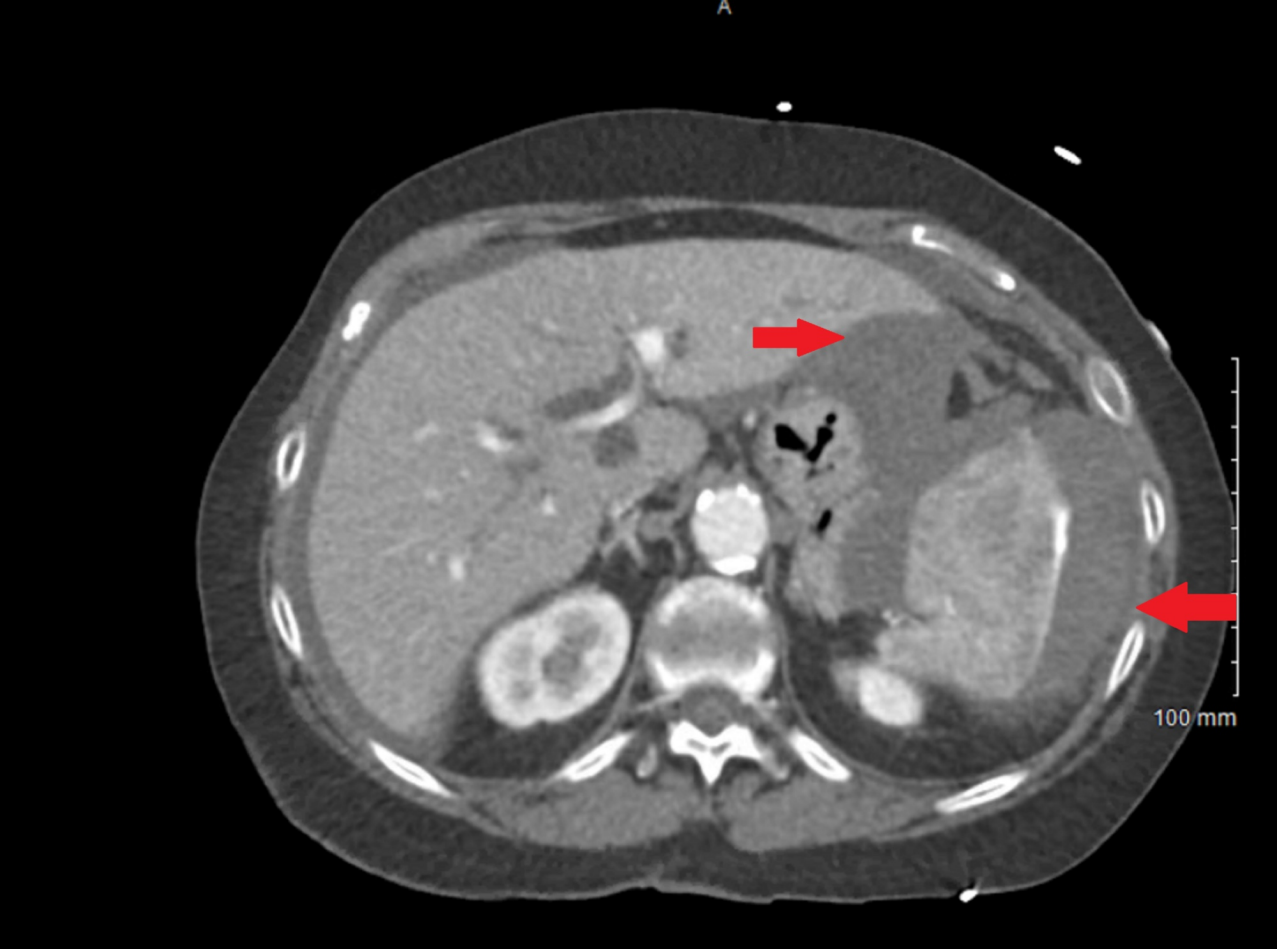
Contrast-enhanced abdominal computed tomography, red arrows showing perisplenic fluid

**Figure 2:**
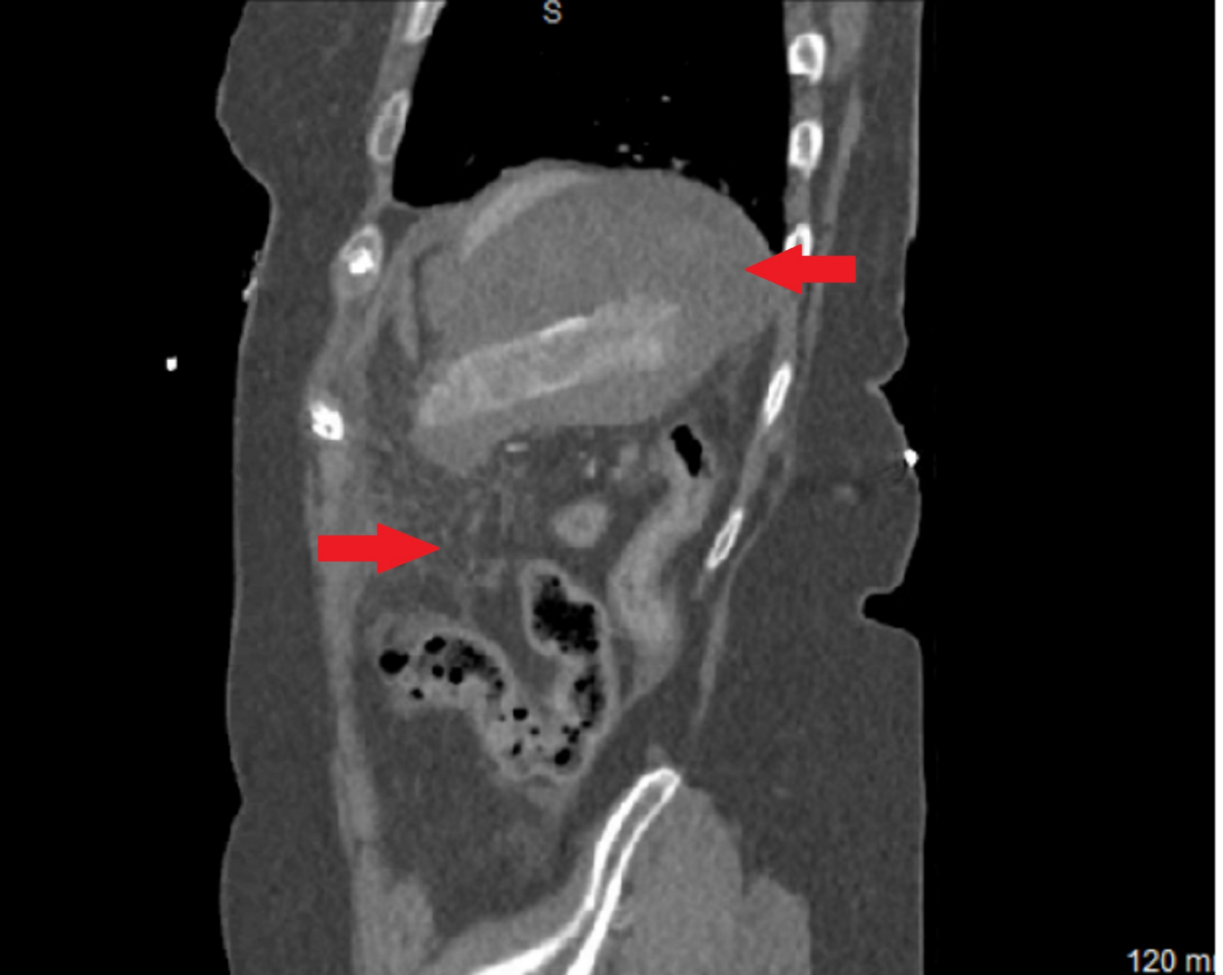
Contrast-enhanced abdominal computed tomography (sagittal view) with red arrows showing perisplenic and peritoneal fluid

**Figure 3:**
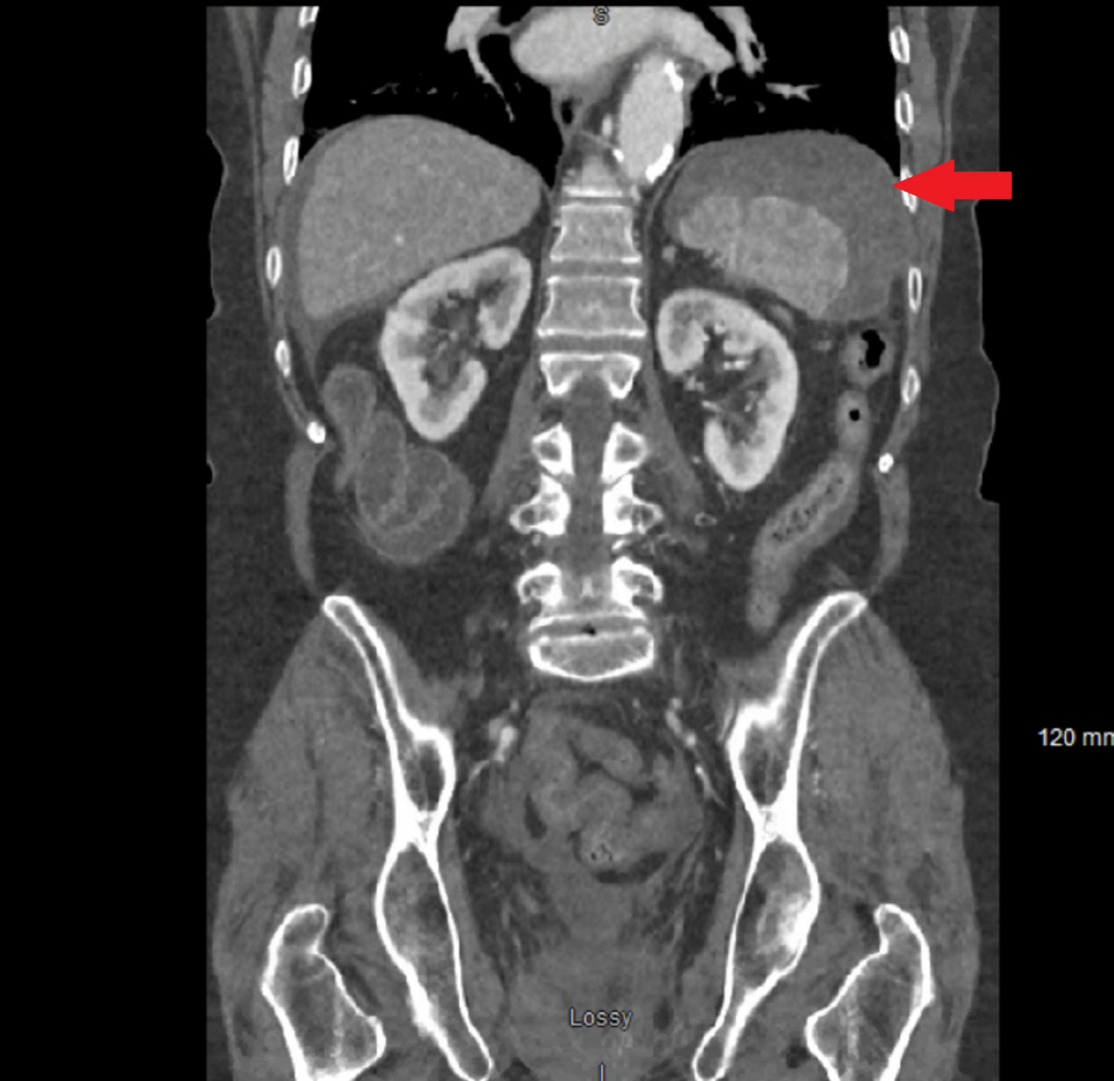
Contrast-enhanced abdominal computed tomography (coronal view) with a red arrow showing perisplenic fluid

The patient underwent an uncomplicated colonoscopy 16 days earlier during which a 1.3 cm polyp was excised. Upon presentation, vital signs were significant for a blood pressure of 96/58 mmHg. Blood work showed a hemoglobin of 7.2 g/dL and an international normalized ratio (INR) of 1.6. There were findings suspicious for splenic rupture on computed tomography (CT) scan of the abdomen (Figures 1-3). The patient received two units of packed red blood cells in the emergency department (ED) and was then taken to the operating room (OR) for emergent exploratory laparotomy. Intraoperatively, the spleen was found to be ruptured and multiples clotted fragments were seen. Therefore, a splenectomy was performed. The patient did not have any complications during or after the procedure and was discharged six days after the splenectomy in stable condition. Appropriate vaccinations were administered upon discharge.

## Discussion

Colonoscopy is done both in the inpatient and outpatient settings. It has been estimated that around 1.3% of the patients who undergo colonoscopy in the outpatient setting later present to the emergency department with complaints of abdominal pain [7]. There are many proposed mechanisms for spleen injury during colonoscopy; these include tension on the splenocolic ligament, the tension on preexisting intra-abdominal adhesions, and direct injury to the spleen during the passage of the colonoscope through the splenic flexure [8]. Keeping in mind the fact that the spleen is a highly vascular organ, intraperitoneal hemorrhage with resultant hypovolemic shock can occur because of splenic rupture due to loss of the circulatory blood volume. Physiologically, it can be speculated that our patient's use of apixaban might have played a contributing role. The use of anticoagulants increases the risk of bleeding and the spleen is no exception. As described in our case, the spleen was observed to be in multiple clotted pieces. This observation raises questions regarding the effects of the blood thinners on the friability of the spleen due to chronic leakiness of blood into the spleen. The presentation of splenic injury is variable. While some patients present with normal vital signs and unremarkable physical examination, others can present with severe abdominal pain and hemorrhagic shock. Most patients present with new-onset abdominal pain within 24 hours of colonoscopy, but delayed presentations have also been reported [9-10]. To our knowledge, only five cases of splenic injury have been previously reported with the time of presentation greater than 16 days post-colonoscopy. CT scan of the abdomen and pelvis is one of the most sensitive and specific methods for the diagnosis of splenic injury. The most recent guidelines of the American Association for the Surgery of Trauma (AAST) suggest that splenic injury patients should be managed non-operatively if found to be hemodynamically stable. Alternative options include splenectomy or surgical embolization [11].

## Conclusions

Our patient's symptoms and location of pain are not typical for splenic injury. With our case, we intend to spread awareness about this not so uncommon but possibly fatal complication of colonoscopy with delayed and atypical symptoms.
